# Time to consider more than just calcium? The impact on protein, riboflavin, vitamin B12 and iodine intake of replacing cows’ milk with plant-based milk-like drinks—an Australian usual intake dietary modelling study

**DOI:** 10.1007/s00394-025-03697-8

**Published:** 2025-05-23

**Authors:** Anita S. Lawrence, Daniel Russo-Batterham, Kim Doyle, Edoardo Tescari

**Affiliations:** 1 Institute for Physical Activity and Nutrition (IPAN), Faculty of Health, Burwood, VIC 3125 Australia; 2 Melbourne Data Analytics Platform, The University of Melbourne, Parkville, VIC 3010 Australia

**Keywords:** Plant-based milk-like drink, Milk alternatives, Riboflavin, Iodine, Vitamin B12, Milk

## Abstract

**Purpose:**

Most plant-based milk-like (PBML) drinks sold in Australia are not fortified with riboflavin, vitamin B12 or iodine. Reduced dairy intake is often recommended for planetary health and the 2013 Australian Dietary Guidelines advise that PBML drinks are a suitable replacement for cows’ milk if calcium fortified. We investigated the likely population-wide impacts on riboflavin, vitamin B12, iodine and protein usual intakes of replacement of cows’ milk with PBML drinks.

**Methods:**

We used computer simulation modelling of data from the 2011–12 National Nutrition and Physical Activity Survey (n = 11,925 persons aged 2 + years). Cows’ milk was replaced with PBML drinks and the likely impacts on usual intakes of riboflavin, vitamin B12, iodine and protein were assessed across eight age groups (National Cancer Institute method). A usual intake below the Estimated Average Requirement (EAR) was defined as inadequate.

**Results:**

Replacement of cows’ milk with unfortified PBML drinks would likely lead to an increased proportion of older women (71 + years) with an inadequate riboflavin intake (from 20 to 31%), of older men and females aged 14 + years with an inadequate vitamin B12 intake (from < 1 to 9%, from 5–8 to 11–17%, respectively), and an increased proportion of males and females (2 + years) with an inadequate iodine intake (from 2 to 5%, from 8 to 16%, respectively). Effects on protein adequacy were more minor except for older adults.

**Conclusion:**

Replacement of cows’ milk with most types of Australian PBML drinks has the potential to adversely impact riboflavin, vitamin B12, iodine and protein intake adequacy within the Australian population and future recommendations should consider all population groups and a range of nutrients, not just calcium.

**Supplementary Information:**

The online version contains supplementary material available at 10.1007/s00394-025-03697-8.

## Introduction

The food system is estimated to be responsible for approximately one third of greenhouse gas emissions [1]. Therefore, there is an urgent need to transition populations to diets that are both healthy and more environmentally sustainable. As plant-based milk-like (PBML) drinks are linked to lower greenhouse gas emissions than cows’ milk [2], influential organisations such as the United Nations Environment Program, EAT Lancet, the UK’s Climate Change Committee and the World Wildlife Fund are recommending substantial reductions in dairy intake [3–7]. Similarly, food procurement policies developed to encourage the transition to more environmentally sustainable dietary habits [8] often advise transitioning away from cows’ milk and towards PBML drinks. For example, the supply of PBML drinks increased by 250% and that of cows’ milk declined by 17% in Copenhagen childcare centres because of this type of policy [9].

Replacement of cows’ milk with PBML drinks is a growing trend in countries such as Australia, with a 55% increase in consumption of PBML drinks and an 8% decrease in cows’ milk in the 5 years prior to 2022–23 [10]. In 2023/24 approximately 24% of Australian adults (18–64 years) consumed PBML drinks, and 39% of Australian households purchased both cows’ milk and PBML drinks in 2022/23 [11].

Australian consumers face a wide range of choices when purchasing unflavoured PBML drinks. A 2022 audit conducted at major Melbourne supermarkets identified 157 different products (55 almond, 60 oat, 28 soy, 11 coconut and 3 rice) [12]. Similarly, a 2023 audit at major supermarkets and local food retailers in the less populous Illawarra region of New South Wales identified 129 different types of unflavoured PBML drinks (40 almond, 39 oat, 27 soy, 5 coconut, 5 rice and 13 ‘other’) [13]. Analysis of the ingredient lists from the almond, oat, soy, coconut and rice milks revealed that although 83% of these were fortified with calcium, only 25% were fortified with riboflavin, 28% with vitamin B12 and 3% with iodine. Apart from soy drink, all types contained significantly less protein than cows’ milk [13].

Despite the diversity of products available and increased proportion of the population consuming them, research on the impact of PBML drinks on health outcomes for children and adults is limited. A 2024 systematic review identified just six studies evaluating the nutritional and growth effects of PBML drinks, concluding that height, body mass index and micronutrient intake were lower in children who consumed PBML drinks than those who consumed cows’ milk [14]. Similarly, a 2023 systematic review of intervention studies in adults identified that most of the studies were focused on soy drink (n = 27), with few conducted with other types of PBML drink (rice: n = 2; almond: n = 1), concluding that the results were too conflicting to draw overall conclusions [15].

The 2013 Australian Dietary Guidelines advise that PBML drinks fortified with at least 100 mg of calcium per 100 ml (e.g. soy, rice or other cereal) can replace cows’ milk in the Australian diet [16]. Likewise, the UK’s Eatwell Guide suggests that unsweetened calcium-fortified dairy alternatives made from soy, coconut or oats count as part of the milk and dairy food group and make ‘good alternatives to dairy products’ [17, 18]. However, dietary modelling studies suggest replacement of cows’ milk with various types of PBML drinks is likely to reduce intakes of various nutrients including riboflavin, vitamin B12 and iodine [19, 20]. Similarly, replacement of cows’ milk by PBML drinks (48% soy, 44% almond and 8% other) in the diets of 12,153 Australians aged 2 + years was predicted to lead to decreased intakes of riboflavin, vitamin B12 and iodine [21]. Additionally, this study assessed the implications of these dietary changes for four different population groups (children 2–3 years, men 19–30 years, women 19–30 years and older adults 71 + years). It highlighted variations in the likely nutritional implications between groups, due to differences in the relative amount of cows’ milk consumed and existing levels of inadequacy in the population group, with children 2–3 years likely to be most impacted [21]. The results confirmed the importance of considering all population groups, particularly young children, women of childbearing age and elderly adults when assessing the impacts of replacing animal source foods with plant-based foods. However, only one day of dietary intake data was used in the analysis, preventing quantification of the impact of the modelled dietary changes on mean usual intakes and the proportion of the population achieving the recommended intake of these nutrients.

The National Cancer Institute (NCI) method of data analysis allows the estimation of usual intake using two days of dietary intake data to enable population assessment of intakes with the Nutrient Reference Values [22, 23]. The vast majority of PBML drinks sold in Australia are not fortified with riboflavin, vitamin B12 and iodine, and PBML drinks other than soy drinks are lower in protein than cows’ milk. Therefore, the aim of this study was to assess the likely population-wide impacts on protein, riboflavin, vitamin B12 and iodine intakes of replacement of cows’ milk with the main categories of PBML drinks available in Australian supermarkets in November 2023.

## Methods

### Data source and preparation

A microsimulation dietary modelling design using microdata from the 2011–12 National Nutrition and Physical Activity Survey (NNPAS) was used for the present study, with reporting consistent with ‘Critical appraisal criteria for methodology and reporting quality assessment dietary simulation modelling’ [24]. The NNPAS is a component of the Australian Health Survey 2011–13 and the NNPAS data were collected between May 2011 and June 2012 from 9,519 private households throughout Australia that were randomly selected using a complex, stratified, multistage probability cluster sampling design, providing a total survey of 12,153 persons aged two years and over, which was benchmarked to the Australian population living in non-Very Remote areas of Australia. Full details have been published elsewhere [25]. As our dietary modelling study was a secondary data analysis, additional ethics approval was not required.

In the NNPAS, dietary intake was assessed by 24 h dietary recalls administered by trained interviewers using the Automated Multiple-Pass Method. All participants completed a single 24-h recall and 64% of them completed a second 24 h recall [26]. As dietary recommendations for females differ with pregnancy and lactation [27], data from the 228 women who reported being in either of these conditions were excluded from the analysis, resulting in data from 11,925 day-1 and 7,585 day-2 24 h dietary recalls being utilised in this analysis. Nutritional composition of the recorded dietary intakes was calculated using complete composition data for protein, riboflavin, vitamin B12 and iodine from 5740 foods and drinks listed in the Australian Food and Nutrition Database (AUSNUT2011-13) [28]**.** In the analyses, the dietary intake data (food only, not supplements) were weighted against population benchmarks for age, sex and area of usual residence.

### Cows’ milk replacement scenarios

In this study, cows’ milk was defined as all food codes named ‘Milk, cow, fluid’ (27 codes detailed in Supplementary Table S1A). To identify cows’ milk consumed as hot drinks (e.g. coffee and tea), the recipes for these products (codes11102001 to 11,803,004,114 codes listed in Supplementary Table S1B) were disaggregated using the AUSNUT 2011–13 Food Recipe File [29].

An audit of unflavoured soy, almond, oat, rice and coconut milk-like drinks available in the major Australian supermarket chains was conducted in November 2023, and the nutrient contents listed on the food labels and ingredient lists were compared with the values listed in the Australian Food Composition Database (Supplementary Table S2). These Australian Food Composition Database values were obtained from analysis of representative composites of the main PBML drinks available for purchase in Australia in 2010 for soy and rice drinks, in 2015 for oat drink and 2018 for almond and coconut drinks [30]. As the food labels on the unflavoured soy, almond, oat, rice and coconut milk-like drinks mostly contained little or no information about the riboflavin, vitamin B12 and iodine contents, and the protein contents were similar to those listed in the Australian Food Composition Database, nutrient composition values from the Australian Food Composition Database were used in our dietary modelling. Table 1 shows the protein, riboflavin, vitamin B12 and iodine content of the 11 main categories of PBML drinks available in Australia [30], and the relative proportion of each nutrient compared with regular-fat cows’ milk, as 73% of cows’ milk consumed in Australia is regular-fat [31].

**Table 1:** Protein, riboflavin, vitamin B12 and iodine content of the main types of plant-based milk-like (PBML) drinks sold in Australia, compared with cows’ milk

			Protein (g/100 ml)		Riboflavin (mg/100 ml)		Vitamin B12 (µg/100 ml)		Iodine (µg/100 ml)	
Type	PBML drink	Product details and AFCD code	AFCD values	Amount compared with regular-fat cows’ milk*	AFCD values	Amount compared with regular-fat cows’ milk*	AFCD values	Amount compared with regular-fat cows’ milk*	AFCD values	Amount compared with regular-fat cows’ milk*
Soy	Drink 1-soy	1. Regular fat, unfortified F008721	3.8	112%	0.031	16%	0	0%	1.4 µg	6%
	Drink 2-soy	2. Regular fat, added calcium F008719	3.8	112%	0.031	16%	0	0%	1.4 µg	6%
	Drink 3-soy	3. Regular fat, added calcium and vitamins A, B1, B2 and B12 F008720	4.5	132%	0.422	224%	0.9	225%	1.4 µg	6%
Almond	Drink 4-almond	4. Added sugar, unfortified F009825	0.5	15%	0.020	11%	0	0%	1.1 µg	5%
	Drink 5-almond	5. Added sugar and calcium F009827	0.5	15%	0.021	11%	0	0%	1.2 µg	5%
	Drink 6-almond	6. Added sugar, added calcium and vitamins B1, B2 and B12 F009828	0.5	15%	0.294	156%	0.4	100%	1.2 µg	5%
	Drink 7-almond	7. No added sugar, added calcium F009826	0.5	15%	0.021	11%	0	0%	1.2 µg	5%
Oat	Drink 8-oat	8. Unfortified F006132	1.4	41%	0	0%	0	0%	1.2 µg	5%
	Drink 9-oat	9. Added calcium F006131	1.4	41%	0	0%	0	0%	1.3 µg	6%
Rice	Drink 10-rice	10. Added calcium F007632	0.3	9%	0	0%	0	0%	5.4 µg	23%
Coconut	Drink 11-coconut	11. Unfortified F009812	0.2	6%	0	0%	0	0%	1.3 µg	6%

*AFCD* Australian Food Composition Database

*Regular fat cows’ milk (AFCD code F005634), Shading indicates plant-based milk-like drinks used in dietary modelling

‘Base cases’ were derived from the NNPAS 2011–12 dietary intake. Table S3 shows the proportion of total protein, riboflavin, vitamin B12 and iodine provided by cows’ milk in the ‘Base case’ for various population groups. In each scenario, the base case was taken and cows’ milk (including that consumed in hot drinks) was replaced on an equal volume basis with a representative type of PBML drink in Australia. The impact of one-for-one replacement scenarios of cows’ milk with the PBML drinks (Scenarios 1 and 2) were modelled using Python Jupyter Notebooks (Anaconda3) [32], SASPy and SAS Studio (SAS Institute Inc). The PBML drinks used in the scenarios were selected to be: 1) as representative as possible in terms of their protein, riboflavin, vitamin B12 and iodine contents of most products available for sale at Australian supermarkets, and 2) as they met the 2013 Australian Dietary Guidelines criteria as a replacement for cows’ milk.

#### Scenario 1: The impact of replacement of cows’ milk with PBML drinks on usual protein intakes

Due to the diversity in protein content between the various types of PBML drinks, the impact of replacing cows’ milk with either a calcium-fortified, medium-protein oat drink (Drink 9), or a calcium-fortified lower-protein rice drink (Drink 10), was explored. The oat drink contained 41% of the protein found in cows’ milk and the rice drink contained 9% of the protein found in cows’ milk (see Table 1 and Supplementary Table S2 for composition details). Eight out of 11 main categories of PBML drinks (around 83% of the PBML drinks available for sale at Australian supermarkets in 2023) contain the same amount or less protein than the oat drink. Two out of 11 main categories of PBML drink (around 8% of the PBML drinks available for sale) contain the same amount or less protein than the rice drink. The oat drink was selected as it is the category of PBML drinks with the most products available for purchase (Supplementary Table S2), and the rice drink was selected as it is the PBML drink with the lowest protein that also meets the Australian Dietary Guideline criteria as a suitable cows’ milk replacement.

#### Scenario 2: the impact of replacement of cows’ milk with PBML drinks on usual riboflavin, vitamin B12 and iodine intakes

In this scenario cows’ milk was replaced with calcium-fortified soy drink (Drink 2) (see Table 1 and Supplementary Table S2 for composition details). This soy drink was selected because most (9/11, 9/11 and 10/11) types of unflavoured soy, almond, oat, rice and coconut milk-like drinks (around 77, 77 and 95% of the products available for sale in Australian supermarkets in 2023) contain this amount or less riboflavin, vitamin B12 and iodine, respectively (Table 1), and therefore the amounts of riboflavin, vitamin B12 and iodine present in this product are representative of the amounts found in most types of Australian PBML drinks.

### Usual intake assessment

For the base case and the scenarios listed earlier, the National Cancer Institute (NCI) method [23, 33, 34] was used to assess the mean usual intake and the percentage of various population groups with an inadequate usual intake of protein, riboflavin, vitamin B12 and iodine. A usual intake below the Estimated Average Requirement (EAR) was used to define inadequate intake [26, 27]. In the NCI usual intake method, pooled intake records are used to estimate a population group’s usual intake distribution, with within-person variation removed and adjustments made for the day of the week the dietary assessment was being conducted.

The method used was similar to that used in the usual intake analysis of the 2011–12 NNPAS. The ‘one-part’/‘amount-only’ NCI model was selected, as the nutrients assessed are consumed nearly every day by nearly everyone. To allow for differences between males and females, the dataset was analysed as three smaller datasets: children aged below nine years, males nine years and over, and females nine years and over. Results were produced for 16 population groups, aligning with the NRV recommendations: 2–3, 4–8, 9–13, 14–18, 19–30, 31–50, 51–70 and 71 + years for males and females separately. Standard errors (SE), relative standard errors (RSE) and margin of errors (MoE) were calculated using the replicate weights method [25]. To determine if differences between the base case and the scenarios were statistically significant, the following formula was used: (x–y)/SE(x–y). Values greater than 1.96 were considered to be statistically significant different [25] and meaningful [35]. Comparison of base case results with previously published data [26] confirmed internal validity.

## Results

### Scenario 1: the impact of replacement of cows’ milk with plant-based milk-like drinks on usual protein intakes

Table 2 shows the potential impacts of replacing cows’ milk with two different PBML drinks, calcium-fortified oat drink (Drink 9) and calcium-fortified rice drink (Drink 10), which contain 41 and 9% respectively, of the protein present in cows’ milk. Overall, for the Australian population (2 + years) usual protein intake would likely remain about the same or decline by 3% if oat drink or rice drink, respectively, is substituted for cows’ milk.

**Table 2:** Scenario 1: Estimated change in mean usual intake of protein and the proportion of the population with a protein intake below the Estimated Average Requirement if cows’ milk is replaced by calcium-fortified oat drink (Drink 9) or calcium-fortified rice drink (Drink 10)

	Australian diet with cows’ milk					Australian diet with oat drink (Drink 8)					Australian diet with rice drink (Drink 10)				
	Usual daily intake		Less than EAR			Usual daily intake		Less than EAR			Usual daily intake		Less than EAR		
	Mean (g/d)	RSE (%)	%	95% MoE (±)	n^a^ (‘000)	Mean (g/d)	RSE (%)	%	95% MoE (±)	n^a^ (‘000)	Mean (g/d)	RSE (%)	%	95% MoE (±)	n^a^ (‘000)
All persons	87		0.9	0.2	193	87		1.2	0.2	256	84		1.4	0.2	300
Males															
2–3 years	61	3	–	–	–	54*	3	–	–	–	51*	3	–	–	0
4–8 years	67	2	–	–	–	63*	3	–	–	–	60*	3	–	–	0
9–13 years	87	3	–	–	–	83	3	0.1	0.2	–	81*	3	0.1	0.4	1
14–18 years	104	3	0.1	0.4	1	100	3	0.5	1.3	3	98*	3	0.6	1.4	4
19–30 years	113	2	0.1	0.2	2	112	2	0.2	0.4	3	109	2	0.2	0.4	4
31–50 years	108	1	0.2	0.3	6	108	2	0.3	0.4	8	106	2	0.3	0.6	11
51–70 years	98	1	0.6	0.7	14	97	2	0.9	0.9	23	95	2	1.2	1.2	28
71 and over	86	2	13.7	5.1	113	84	2	17.6	8.4	145	82*	2	20.0	8.1	165
All males	99		1.3	0.3	136	99		1.7	0.3	183	95		2.0	0.4	212
Females															
2–3 years	53	3	–	–	–	48*	2	–	–	–	45*	3	–	–	–
4–8 years	59	2	–	–	–	56*	2	–	–	–	54*	2	–	–	–
9–13 years	74	3	–	–	–	71*	2	–	0.2	–	69*	2	–	0.2	–
14–18 years	76	3	0.3	0.5	2	74	3	0.3	0.8	2	73	3	0.4	0.8	3
19–30 years	77	2	0.3	0.6	5	76	2	0.5	1.0	8	75	2	0.5	1.1	9
31–50 years	79	2	0.2	0.3	6	80	2	0.2	0.4	6	78	2	0.2	0.4	7
51–70 years	78	2	0.3	0.3	7	78	2	0.3	0.5	7	76	2	0.4	0.7	9
71 and over	73	2	3.8	2.5	37	71	2	5.1	2.7	50	69*	2	6.2	2.7	0
All females	75		0.6	0.2	58	74		0.7	0.2	74	73		0.8	0.2	88

*RSE* Relative Standard Error, *MoE* Margin of Error

*Significantly different to usual Australian diet with cows’ milk;—nil or rounded to zero

^**a**^Calculated using Australian population estimates from the 2011–12 Australian Health Survey: Usual Nutrient Intakes

**Fig. 1:**
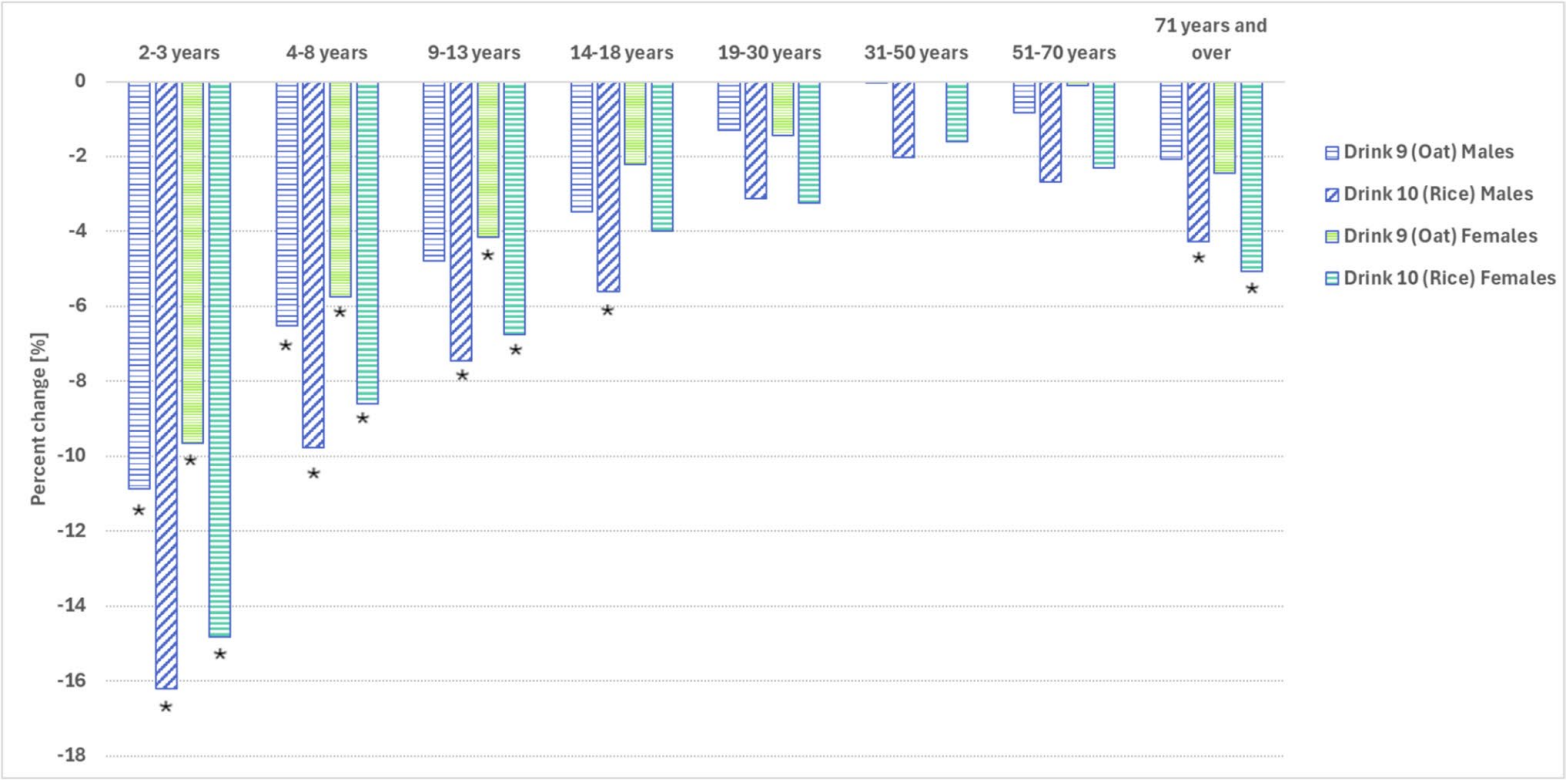
Percentage change in usual protein intake if calcium-fortified oat drink (Drink 9) or calcium-fortified rice drink (Drink 10) is substituted for cows’ milk (Scenario 1). *Statistically significant change

In terms of the impact on the 16 different population groups, usual protein intakes would likely be reduced by 7–11% in boys aged 2–8 years and by 4–10% in girls aged 2–13 years if calcium-fortified oat drink is substituted for cows’ milk (Fig. 1). Replacement of cows’ milk with calcium-fortified rice drink would likely reduce usual protein intake by 6–16% in boys aged 2–18 years, by 7–15% in girls aged 2–13 years and by 4–5% in adults aged 71 years and over (Fig. 1).

**Fig. 2:**
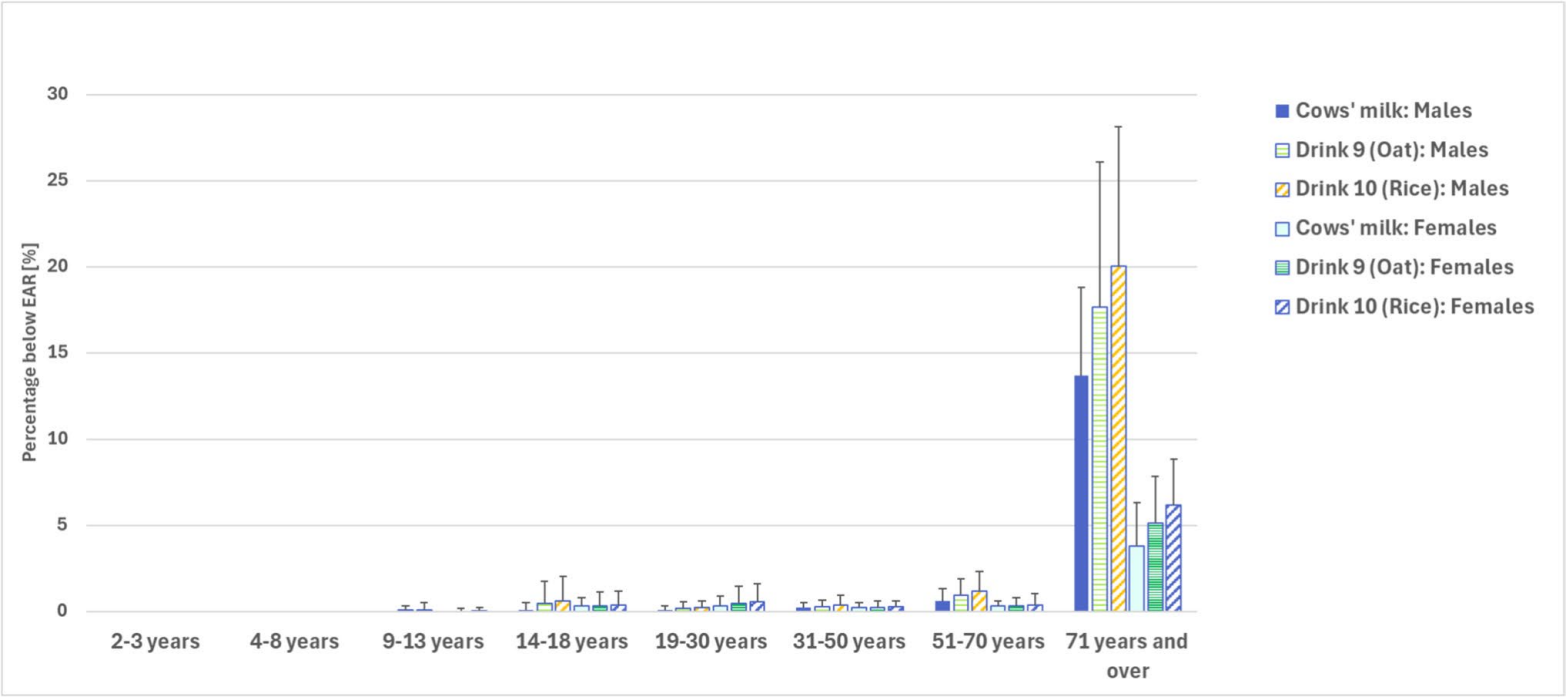
The implications of replacing cows’ milk with calcium-fortified oat drink (Drink 9) or calcium-fortified rice drink (Drink 10) on the proportion of the population with a protein intake below the Estimated Average Requirement (EAR)

Despite these estimated decreases in protein intake, protein intake would likely remain adequate (equal to or above the EAR) for at least 99% of males aged 2–50 years and females aged 2–70 years (Table 2). Overall, 20% (MoE 8.1%) of older men (71 + years) and 6% (2.7%) of older women (71 + years) would likely have an inadequate protein intake if they consumed calcium-fortified rice drink in place of cows’ milk, however, these values are not statistically significantly different to the baseline values of 14% (5.1%) and 4% (2.5%), respectively (Fig. 2).

### Scenario 2: The impact of replacement of cows’ milk with plant-based milk-like drinks on usual riboflavin, vitamin B12 and iodine intakes

#### Riboflavin

**Fig. 3:**
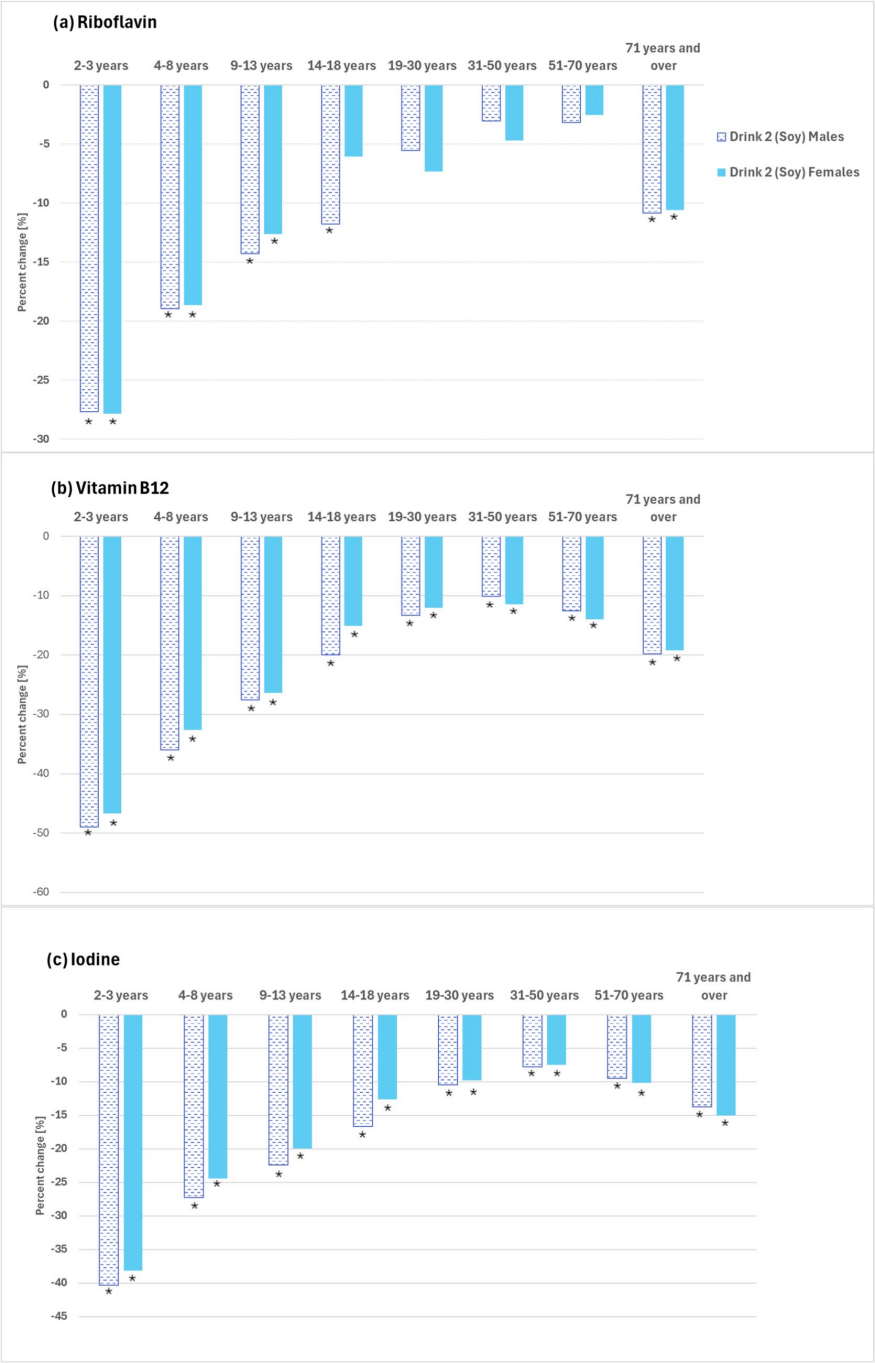
Percentage changes in usual **a** riboflavin, **b** vitamin B12, **c** iodine intakes if calcium-fortified soy drink (Drink 2) unfortified with riboflavin, vitamin B12 or iodine is substituted for cows’ milk. *Statistically significant

Overall, replacement of cows’ milk with non-riboflavin-fortified soy drink (Drink 2) would likely lead to a non-significant 11% reduction in usual riboflavin intake for the Australian population (aged 2 years and over) (Table 3). Nine out of the 11 main types of PBML drink (around 77% of the products available for sale in Australian supermarkets in 2023) contain a similar amount of riboflavin as this soy drink, or less (Table 1). However, replacing cows’ milk with non-riboflavin-fortified soy drink would likely lead to meaningful reductions in usual intakes of riboflavin by 12 to 28% in boys aged 2–18 years, by 13–28% in girls aged 2–13 years and by 11% in older adults aged 71 years and over (Fig. 3a and Table 3).

**Table 3:** Scenario 2: Estimated change in mean usual intake of riboflavin and the proportion of the population with a riboflavin intake below the Estimated Average Requirement if cows’ milk is replaced by calcium-fortified soy drink (Drink 2) not fortified with riboflavin

	Australian diet with cows’ milk					Australian diet with soy drink (Drink 2)				
	Usual daily intake		Less than EAR			Usual daily intake		Less than EAR		
	Mean (µg/d)	RSE (%)	%	95% MoE (±)	n^a^ (‘000)	Mean (µg/d)	RSE (%)	%	95% MoE (±)	n^a^ (‘000)
All persons	1.9		6.9	0.5	1453	1.7		9.9	0.5	2093
Males										
2–3 years	1.9	3.8	–	–	0	1.4*	4.0	–	–	0
4–8 years	1.9	2.9	–	–	0	1.5*	3.4	–	–	0
9–13 years	2.1	4.2	1.0	1.2	7	1.8*	4.2	3.2	2.9	22
14–18 years	2.2	4.3	4.6	3.5	34	1.9*	4.3	9.7	5.6	70
19–30 years	2.3	3.2	3.4	2.4	67	2.2	3.6	5.0	3.7	99
31–50 years	2.2	2.1	4.4	1.9	135	2.1	2.4	5.8	2.5	179
51–70 years	1.9	1.7	8.7	3.0	208	1.8	2.4	12.3	4.2	294
71 and over	1.9	3.4	20.3	6.2	167	1.7*	3.2	30.2	9.9	248
All males	2.1		5.8	0.6	618	1.9		8.5	0.7	913
Females										
2–3 years	1.7	3.3	–	–	0	1.2*	3.5	–	–	0
4–8 years	1.7	3.2	–	–	0	1.4*	3.3	–	–	0
9–13 years	1.8	3.6	2.9	1.9	19	1.6*	3.7	6.5	4.5	44
14–18 years	1.6	4.1	8.4	4.5	58	1.5	5.0	13.4	6.9	92
19–30 years	1.7	3.4	7.7	4.1	132	1.6	3.6	10.7	4.1	183
31–50 years	1.7	1.6	7.8	2.3	228	1.6	2.2	9.5	2.7	279
51–70 years	1.6	2.0	8.1	2.7	200	1.6	2.4	11.4	4.1	280
71 and over	1.6	3.3	20.3	5.8	199	1.4*	3.4	30.8*	6.3	302
All females	1.7		8.0	0.7	835	1.5		11.3	0.8	1180

*RSE* Relative Standard Error, *MoE* Margin of Error

*Significantly different to usual Australian diet with cows’ milk;—nil or rounded to zero

^**a**^Calculated using Australian population estimates from the 2011–12 Australian Health Survey: Usual Nutrient Intakes

**Fig. 4:**
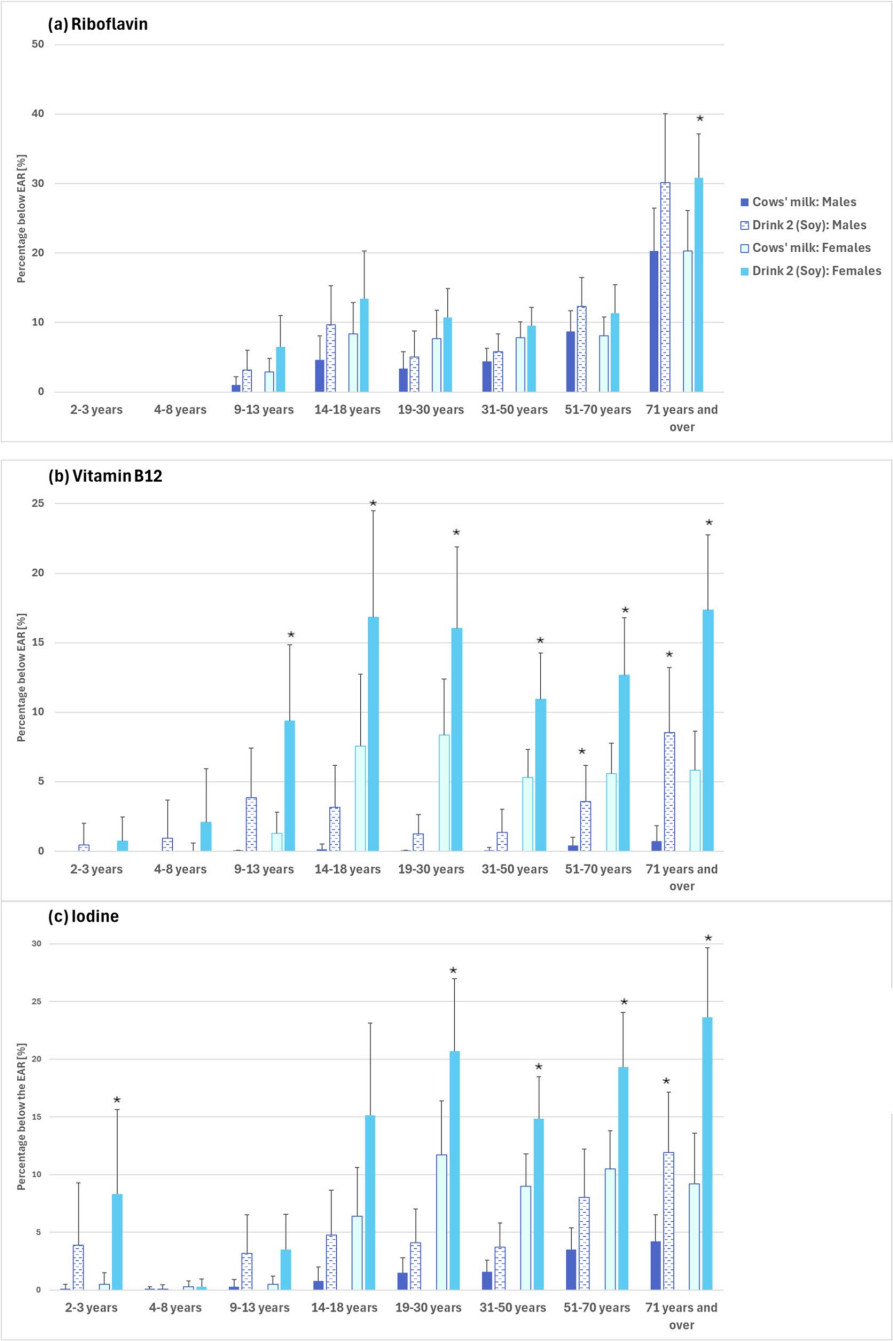
The implications of replacing cows’ milk with calcium-fortified soy drink (Drink 2) not fortified with riboflavin, vitamin B12 or iodine on the proportion of the population with usual **a** riboflavin, **b** vitamin B12, **c** iodine intake below the Estimated Average Requirement (EAR). *Statistically significant difference between usual diet with cows’ milk and usual diet with calcium-fortified soy drink

Even with these reductions, virtually all children aged 2–8 years and at least 95% of children aged 9–13 years would likely continue to have an adequate intake of riboflavin (Fig. 4a and Table 3). In contrast, replacement of cows’ milk with non-riboflavin-fortified soy drink would likely increase the proportion of women aged 71 + years with an inadequate riboflavin intake from 20 to 31%.

#### Vitamin B12

Overall, replacement of cows’ milk with non-vitamin B12-fortified soy drink (Drink 2) would likely lead to a meaningful decrease to usual vitamin B12 intakes, reducing them overall by 16% for the Australian population (aged 2 + years) (Table 4). Nine out of the 11 main types of PBML drink (around 77% of the products available for sale in Australian supermarkets in 2023), like this soy drink, contain no vitamin B12 (Table 1). Usual intakes of vitamin B12 would likely decline significantly for all 16 population groups, with the lowest reduction (10%) predicted for men aged 31–50 years and the greatest reduction (49%) for boys aged 2–3 years (Fig. 3b, Table 4).

**Table 4:** Scenario 2: Estimated change in mean usual intake of vitamin B12 and the proportion of the population with a vitamin B12 intake below the Estimated Average Requirement if cows’ milk is replaced by calcium-fortified soy drink (Drink 2) not fortified with vitamin B12

	Australian diet with cows’ milk					Australian diet with soy drink (Drink 2)				
	Usual daily intake		Less than EAR			Usual daily intake		Less than EAR		
	Mean (µg/d)	RSE (%)	%	95% MoE (±)	n^a^ (‘000)	Mean (µg/d)	RSE (%)	%	95% MoE (±)	n^a^ (‘000)
All persons	4.4		2.7	0.3	578	3.7		7.4*	0.5	1551
Males										
2–3 years	3.8	4.2	–	–	0	1.9*	5.5	0.5	1.6	1
4–8 years	3.5	3.4	–	–	0	2.2*	4.5	1.0	2.7	7
9–13 years	4.5	4.3	–	–	0	3.3*	4.6	3.9	3.6	27
14–18 years	5.4	3.6	0.1	0.4	1	4.3*	3.9	3.2	3.0	23
19–30 years	5.6	2.9	–	–	0	4.9*	3.5	1.2	1.4	25
31–50 years	5.4	2.3	0.1	0.2	3	4.9*	2.7	1.3	1.7	41
51–70 years	4.8	2.3	0.4	0.6	10	4.2*	2.7	3.6*	2.6	86
71 and over	4.5	2.8	0.8	1.1	7	3.6*	3.2	8.5*	4.7	70
All males	5.0		0.2	0.1	20	4.2		2.6*	0.4	281
Females										
2–3 years	3.3	3.8	–	–	0	1.8*	4.7	0.7	1.7	2
4–8 years	3.1	3.0	0.2	0.6	1	2.1*	4.0	2.1	3.8	14
9–13 years	3.7	4.4	1.7	1.5	11	2.8*	3.9	9.4*	5.5	63
14–18 years	3.6	4.2	7.7	5.2	53	3.1*	4.9	16.8*	7.7	116
19–30 years	3.6	3.4	8.3	4.0	142	3.1*	3.9	16.0*	5.8	274
31–50 years	3.9	2.2	5.5	2.0	161	3.4*	2.9	10.9*	3.3	320
51–70 years	3.8	2.7	5.4	2.2	133	3.3*	3.1	12.7*	4.1	312
71 and over	3.8	3.4	5.8	2.8	57	3.1*	3.5	17.3*	5.4	170
All females	3.7		5.4	0.6	558	3.1		12.2*	0.8	1271

*RSE* Relative Standard Error, *MoE* Margin of Error

*Significantly different to usual Australian diet with cows’ milk;—nil or rounded to zero

^**a**^Calculated using Australian population estimates from the 2011–12 Australian Health Survey: Usual Nutrient Intakes

Despite the predicted meaningful decline in vitamin B12 intakes in males, over 95% of males aged 2–70 years would likely maintain an adequate dietary intake (Fig. 4b). In contrast, the proportion of older men (71 + years) with an inadequate intake would likely increase from less than 1 to 8.5% (MoE 4.7%).

For females, the predicted decline in usual mean vitamin B12 intake would likely lead to an increased proportion of females aged 9 + years consuming an inadequate vitamin B12 intake (Fig. 4). The proportion of females aged 14–70 years with an inadequate vitamin B12 intake would likely double (to 11–17%), compared with current levels, and for women aged 71 + years, this proportion would likely triple (17%). The proportion of girls aged 9–13 years with an inadequate B12 intake would likely increase by sevenfold increase (to 9%) (Fig. 4b, Table 4).

#### Iodine

Overall, there would likely be a meaningful 12% (21 µg/d) decline in mean iodine intake for the Australian population (persons 2 + years) if cows’ milk is replaced with a non-iodine-fortified PBML drink such as the soy drink (Drink 2) used in this modelling (Table 5). The iodine content of this type of PBML drink is representative, as ten out of the 11 main types of PBML drinks (around 95% of the products available for sale in Australian supermarkets in 2023) contain a similar amount of iodine or less (Table 1). Replacement of cows’ milk with this type of non-iodine-fortified PBML drink would likely lead to mean usual iodine intakes that are significantly lower for males and females of all ages, with the decreases varying according to age group and sex. The smallest predicted decrease being 7% for women aged 31–50 years and the greatest a 40% decrease in boys aged 2–3 years (Table 5 and Fig. 3c).

**Table 5:** Scenario 2: Estimated change in mean usual intake of iodine and the proportion of the population with an iodine intake below the Estimated Average Requirement if cows’ milk is replaced by calcium-fortified soy drink (Drink 2) not fortified with iodine

	Australian diet with cows’ milk					Australian diet with soy drink (Drink 2)				
	Usual daily intake		Less than EAR			Usual daily intake		Less than EAR		
	Mean (µg/d)	RSE (%)	%	95% MoE (±)	n^a^ (‘000)	Mean (µg/d)	RSE (%)	%	95% MoE (±)	n^a^ (‘000)
All persons	171		5.1	0.4	1069	150		10.4*	0.5	2,204
Males										
2–3 years	157	4	0.1	0.4	0	94*	3	3.9	5.4	12
4–8 years	164	2	0.1	0.2	1	119*	3	0.1	0.4	1
9–13 years	190	4	0.3	0.6	2	147*	4	3.2	3.3	22
14–18 years	205	4	0.8	1.2	6	171*	4	4.8	3.9	35
19–30 years	202	2	1.5	1.3	30	181*	3	4.1	2.9	81
31–50 years	200	2	1.6	1.0	49	184*	2	3.7	2.1	115
51–70 years	182	2	3.5	1.9	84	165*	2	8.0	4.2	192
71 and over	178	3	4.2	2.3	35	154*	3	11.9*	5.3	98
All males	191		1.9	0.3	206	167		5.2*	0.6	554
Females										
2–3 years	141	3	0.5	1.0	1	87*	3	8.3*	7.3	24
4–8 years	148	3	0.3	0.5	2	112*	2	0.3	0.7	2
9–13 years	169	3	0.5	0.7	3	135*	3	3.5	3.0	24
14–18 years	153	4	6.4	4.2	44	134*	5	15.1	8.0	104
19–30 years	146	3	11.7	4.7	200	132*	3	20.7*	6.3	354
31–50 years	152	1	9.0	2.8	263	141*	2	14.9*	3.6	435
51–70 years	149	2	10.5	3.3	259	134*	2	19.3*	4.7	476
71 and over	151	3	9.2	4.4	90	128*	2	23.6*	6.0	231
All females	151		8.3	0.7	863	132		15.9*	0.9	1,650

*RSE* Relative Standard Error, *MoE* Margin of Error

*Significantly different to usual Australian diet with cows’ milk;—nil or rounded to zero

^**a**^Calculated using Australian population estimates from the 2011–12 Australian Health Survey: Usual Nutrient Intakes

These predicted declines in mean usual intake would likely lead to a significant increase (from 5% with cows’ milk to 10% with PBML drink) in the prevalence of inadequate iodine intake for the Australian population (2 + years) (Table 5). For males (2 + years), prevalence of inadequate iodine intake would likely increase from 2 to 5% and for females (2 + years) prevalence would increase from 8 to 16%. On a population group basis, the proportion of girls aged 2–3 years and women aged 19 + years not meeting iodine recommendations would increase (Fig. 4c). Approximately 1.7–1.8 more women between the ages of 19 and 70 years and approximately 2.6 times as many women aged 71 + years would likely have an inadequate usual iodine intake. Men aged 71 + years would also be significantly more likely to have an inadequate dietary iodine intake (an increase from 4 to 12%), following a switch from cows’ milk to non-iodine-fortified PBML drinks (Table 5).

## Discussion

As most of the types of PBML drinks available in Australian supermarkets in November 2023 were not fortified with riboflavin, vitamin B12 or iodine, and PBML drinks other than soy drink are lower in protein than cows’ milk, we undertook dietary modelling to estimate the implications of replacement of cows’ milk with these types of PBML drinks within Australian diets, focusing on:1) the theoretical impact on mean usual intakes and 2) achievement of dietary recommendations for these nutrients, for the population as a whole and for 16 different population groups. The results of this dietary modelling indicate that, within the Australian diet, replacement of cows’ milk with PBML drinks unfortified with riboflavin, vitamin B12 or iodine (most types of plant-based milk-like drinks) would likely lead to meaningful decreases in the usual dietary intakes of these nutrients for some or all population groups. Mean usual protein intakes would also likely decline with oat drink in children aged 2–8 years, and with rice drink in children aged 2–13, adolescent boys and adults 71 + years of age.

The results of this study also indicate that replacement of cows’ milk with non-riboflavin-fortified, non-vitamin B12-fortified and non-iodine-fortified PBML drinks would likely lead to an increased proportion of older women (71 + years) with an inadequate intake of riboflavin (20% with cows’ milk vs 31% with PBML drink), an increased proportion of older men (71 + years) and females aged 14 + years with an inadequate intake of vitamin B12 (< 1% with cows’ milk vs 9% with PBML drink and 5–8% with cows’ milk vs 11–17% with PBML drink, respectively) and an increased proportion of males and females with an inadequate dietary iodine intake (2% with cows’ milk vs 5% with PBML drink, and 8% with cows’ milk vs 16% with PBML drink, respectively). In contrast, although PBML drinks other than soy drink tend to be lower in protein than cows’ milk, the predicted impacts of replacement of cows’ milk by low-protein PBML drinks were more minor for most of the population, and protein adequacy is less likely to be compromised other than for older adults (71 + years).

Several PBML drink for cows’ milk substitution modelling studies have also reported likely decreases in intakes of some or all of the following nutrients: riboflavin, vitamin B12, iodine and protein [20, 21, 36]. As these previous studies considered the impact of these dietary changes on aggregated mean intakes, they were unable to determine the impacts of these changes in terms of achievement of recommended intakes. By using the NCI method to assess usual intakes, the current study provides estimates of how these changes in nutrient intake will likely affect the proportions of the Australian population with adequate and inadequate nutrient intakes [22].

Results of the current study are consistent with those of Nicol and colleagues [35], who also used the NCI method in dietary modelling, concluding that replacement of cows’ milk with PBML drinks, particularly those not fortified with iodine, has the potential to adversely affect population iodine intakes in the UK. Nicol and colleagues’ dietary modelling study using UK dietary intakes and PBML drinks revealed a similar pattern of projected declines in mean usual iodine intakes when cows’ milk was replaced with unfortified PBML drinks to those observed in the present Australian study, with the greatest predicted percentage declines in young children (1.5–3 years) and substantial but lower declines among older children, adolescent girls and women of reproductive age.

Declining iodine intakes are a growing concern in developed countries such as the US, UK and Australia [37]. Results of the current study, a predicted 12% decline in usual mean iodine intake and doubling in the prevalence of inadequate iodine intake, support the growing awareness of the need to consider the potential impact on iodine intakes when replacing cows’ milk with most types of PBML drinks [35, 37–39]. Adequate iodine intake is important at all stages of life as it is a co-factor for thyroid hormones (thyroxine and triiodothyronine), and is critical for growth and development and liver, kidney, muscle, brain and central nervous system functioning [37, 38].

Iodine is particularly important for the brain and cognitive development of foetuses, neonates and infants, and women are encouraged to achieve their recommended iodine intake, particularly prior to and during pregnancy and breast feeding [40, 41]. With PBML drink consumers shown to have lower urinary iodine concentrations than cows’ milk consumers [39] and 18% of Australian women of childbearing age classified as iodine deficient (less than 50 µg/L) in the 2011–12 Australia Health Survey [42], the current results indicating that rates of inadequate iodine intake would likely increase in females aged 14 to 50 years by 66–136% (depending on the age group), are concerning.

The results of the present study also highlight the need for greater consideration of vitamin B12 intakes when PBML drink that is not fortified with vitamin B12 replaces cows’ milk, for the population as a whole, and particularly for females of childbearing age and older people. Vitamin B12 is needed for the synthesis of blood cells and brain nerve tissue, and low vitamin B12 status has been reported to be associated with an increased risk of neuropsychiatric and neurological disorders, with frequently reported signs of B12 deficiency including ‘brain fog’, memory problems, headaches, mood swings, depression, fatigue and muscle weakness [43, 44].

The present results, predicting that 11–17% of females of childbearing age (compared with 6–8%) would likely have an inadequate dietary vitamin B12 intake if cows’ milk is replaced by non-B12 fortified PBML drinks, is a particular concern. During pregnancy, adequate vitamin B12 status is important for foetal neural myelination, brain and cognitive development and a low vitamin B12 status has been associated with developmental anomalies and low birth weight [44].

Malabsorption and inadequate dietary intake are two of a number of known factors contributing to vitamin B12 deficiency in older people, a condition affecting approximately 20% of people over 60 years of age [45]. Our results, a predicted increase in prevalence of inadequate intake from 1 to 9% and from 6 to 17% in older men and women, respectively, highlight the need for careful monitoring of the vitamin B12 status of older Australians (71 + years) who replace cows’ milk with unfortified PBML drinks.

Another significant finding of the present study is the predicted decline in usual mean intakes of riboflavin in older adults and the increase in prevalence of inadequate intake by older women (71 + years) (from 20 to 31%) if PBML drink that is not fortified with riboflavin is consumed in place of cows’ milk. This is important to address as dietary riboflavin may be important for cognitive functioning in older adults, likely through its anti-inflammatory and anti-oxidative properties [46–48].

Although our dietary modelling predicts that replacement of cows’ milk with rice drink would likely lead to a 3% decline in overall protein intake for the Australian population overall, the predicted impacts would not be uniform for all population groups, with children, adolescents and older people likely to be most affected. In addition to protein quantity, protein quality is also an important consideration as PBML drinks generally have poorer protein quality than cows’ milk, due to at least one limiting amino acid (e.g. lysine for grains and sulphur-containing amino acids for legumes), a metric mostly not considered in dietary modelling substitution studies [49]. The implications of the predicted decline in usual protein intake, particularly for children, adolescents and older people, are not fully understood but may, according to the diet protein leverage hypothesis, and depending on intake of other constituents of the diet, lead to a compensatory energy intake and even increased obesity rates [50]. At present, PBML drinks containing less protein than cows’ milk are required to carry a label advising that for children under 5 years of age, the product is not suitable as a complete milk replacement [51]. Our results suggest that consideration be given to extending this advice to older adults (71 + years), as this population group is the most likely to have an inadequate protein content, and switching from cows’ milk to rice drink would likely significantly reduce their usual mean protein intake.

To date, much of the focus relating to the nutritional composition of PBML drinks has rightly focused on their calcium and protein content. For example, the 2013 Australian Dietary Guidelines, which provide advice for the general healthy population, recommend that any PBML drinks can replace cows’ milk in the Australian diet (e.g. soy, rice or other cereal) if fortified with at least 100 mg of calcium per 100 ml [16]. Approximately 80% of PBML drinks are fortified with calcium [13], and protein advisory labels are mandated on low-protein PBML drinks. Overall, the results of the present study suggest that there is a need for renewed consideration of population riboflavin, vitamin B12 and iodine intakes when providing advice about the types of PBML drinks suitable to replace cows’ milk. Our results also highlight the need for careful consideration of the potential detrimental nutritional impacts of the current (2013) Australian Dietary Guidelines advice for population groups such as young children, women of childbearing age and older people aged 71+ years.

There are three main ways that inadequate dietary intakes of riboflavin, vitamin B12 and iodine could be addressed and the advantages and disadvantages of each would need to be carefully considered prior to implementation. Firstly, mandatory fortification of PBML drinks could be introduced to ensure that PBML drinks contain similar amounts of these nutrients as cows’ milk. Although the Australia New Zealand Food Standards Code permits voluntary fortification [52], the present study and others have shown that most of the PBML drinks currently sold in Australia contain little fortification other than calcium [13]. Second, through extension of the current advisory labelling (that PBML drinks containing less protein than cows’ milk are not suitable as a complete milk replacement for children under 5 years [51]) to also include advice about iodine for females, vitamin B12 for females aged 14+ and men aged 71+, and riboflavin for women aged 71+ years. Third, by public health messages focused on encouraging users of PBML drinks to consume alternative sources of these nutrients.

Calls have previously been made in the US for clear standards of identity for PBML drinks, including a minimum content of protein, vitamin B12 and riboflavin [53]. Similarly, Nicol and colleagues [35] determined fortification of PBML drinks with ≥ 22.5 and < 45 µg/100 mL of iodine would likely minimize the impact of replacing cows’ milk with PBML drinks in the UK and urged public health messages to signpost alternative sources of iodine. Potential public health risks related to inadequate or excessive intakes of certain nutrients from the growing consumption of plant-based foods mimicking animal-based products were raised at the Codex Alimentarius Committee on Nutrition and Foods for Special Dietary Uses in 2023 and a proposal for new work was to be developed [54]. Globally, it has been estimated that more that 68, 55 and 39% of the population consume an inadequate intake of iodine, riboflavin and vitamin B12, respectively [55] so recommendations to replace cows’ milk with PBML not fortified with these nutrients may be a worldwide concern.

This present study has good external validity because: (1) the dietary modelling used data from the latest Australian national nutrition survey which included all population groups (other than infants/children under 2 years of age and pregnant/lactating women), (2) the modelling was based on actual recorded dietary intakes from 11,925 individuals (including cows’ milk consumed as a component of hot drinks such as tea and coffee), rather than computer generated optimised dietary intakes or intakes based on relatively imprecise national balance sheet data, (3) recent information about the types and composition of PBML drinks sold in Australia informed the modelling, (4) the potential nutritional impacts of replacement of cows’ milk with individual types of PBML drinks, rather than an ‘average’ of fortified and non-fortified PBML drinks, was modelled. These factors are important, as the nutritional composition of PBML drinks vary widely and the popularity of the different types of PBML drinks has varied in recent years [31].

The present study highlights the need for continual monitoring and surveillance of the Australian population as food habits and the food supply change. Other aspects of dietary intake are likely to have changed since the 2011/12 NNPAS. According to annual apparent consumption surveys undertaken between 2018 and 19 and 2022–23, the most pronounced changes in per capita daily consumption were for milk products (− 39 kJ), fats and oils (− 38 kJ), and confectionary (+ 42 kJ) [10]. Although adjustments for the non-cows’ milk dietary changes were unable to be made in the modelling, fats, oils and confectionery are not major sources of riboflavin, vitamin B12, iodine or protein so would be unlikely to impact on our results. Overall protein intake was stable between 2018–19 and 2022–23 [10]. All surveys are prone to sampling and non-sampling errors and dietary intake is prone to under reporting of food intake. When people substitute PBML drink for cows’ milk, they may also change from dairy yogurt and cheese to plant-based yogurt and cheese and consume plant-based meat rather than animal-source meat as in 2023, 39% of Australian adults were actively trying to reduce meat consumption and 13% of adults in 2023/24 regularly consumed meat alternatives [11]. The nutritional impacts of reductions in other animal-source foods within the diet were not explored in the present study. Similarly, intake of other nutrients, such as vitamin D, phosphorus and zinc was beyond the scope of the present study. Consumption of dietary supplements containing riboflavin, vitamin B12 and iodine was not considered, as the 2014–15 National Health Survey indicated that use was rare other than in the form of multi-vitamins, which were consumed by only 17.5% of adults [56] and frequently did not contain these nutrients [57]. Future studies are needed to assess the potential impact of different levels of riboflavin, vitamin B12 and iodine fortification of PBML drinks for all age/sex groups of the Australian population.

## Conclusions

According to the Food and Agriculture Organization (FAO) of the United Nations and the World Health Organization (WHO), one of the principles of a healthy diet is the provision of ‘enough essential nutrients to prevent deficiencies and promote health, without excess’ [58]. Therefore, recommendations about transitioning from cows’ milk to PBML drinks should consider the nutritional composition of the PBML drinks available and existing nutritional inadequacies of the population, particularly in relation to children, older adults and women of childbearing age. The results of this study highlight the need for further monitoring and surveillance of Australian diets, the Australian population and the nutritional composition of PBML drinks available for sale, with a particular focus on iodine intakes and status in females, riboflavin intakes in women aged 71 + years, vitamin B12 intakes in older men and females aged 14 + years, and protein intakes in older adults. Consideration should also be given to making most types of PBML drinks more nutritionally similar to cows’ milk and/or increasing awareness of the nutritional differences while also promoting consumption of alternative food sources of riboflavin, vitamin B12 and iodine.

## Supplementary Information

Below is the link to the electronic supplementary material.

Supplementary file1 (DOCX 41 KB)

## Data Availability

The data used in this research were sourced from the following publicly available dataset: Microdata and Table Builder: Australian Health Survey: Nutrition and Physical Activity – Data from the National Nutrition and Physical Activity Survey 2011–12 component of the Australian Health Survey 2011–13 https://www.abs.gov.au/statistics/microdata-tablebuilder/available-microdata-tablebuilder/australian-health-survey-nutrition-and-physical-activity.
