# Supplementary material for: Time to consider more than just calcium? The impact on protein, riboflavin, vitamin B12 and iodine intake of replacing cows’ milk with plant-based milk-like drinks—an Australian usual intake dietary modelling study
Source: Eur J Nutr. 2025 May 23;64(4):182. doi: 10.1007/s00394-025-03697-8 (PMC12102094; doi:10.1007/s00394-025-03697-8)
Supplement: Supplementary file 1 — Supplementary file1 (DOCX 41 KB) [file 394_2025_3697_MOESM1_ESM.docx]

**Supplementary materials**

**Table S1** Details of the cows’ milk that was replaced by plant-based milk-like drinks in the dietary modelling

1. Codes for the cows’ milk (no disaggregation required)

| **AFCD code** | **AFCD name** |  |  |  |  |
| --- | --- | --- | --- | --- | --- |
| 19101001 | Milk, cow, fluid, regular fat (~3.5%) |  |  |  |  |
| 19101002 | Milk, cow, fluid, regular fat (~3.5%), A2 |  |  |  |  |
| 19101003 | Milk, cow, fluid, regular fat (~3.5%), organic | |  |  |  |
| 19101004 | Milk, cow, fluid, regular fat (~3.5%), raw |  |  |  |  |
| 19101005 | Milk, cow, fluid, lactose free, regular fat (~3.5%) | |  |  |  |
| 19101006 | Milk, cow, fluid, regular fat (~3.5%), not further defined | | |  |  |
| 19101007 | Milk, cow, fluid, prepared from dry powder, regular fat, standard dilution | | | |  |
| 19101008 | Milk & water, regular fat cow's milk & tap water | |  |  |  |
| 19103001 | Milk, cow, fluid, reduced fat (1%) |  |  |  |  |
| 19103002 | Milk, cow, fluid, reduced fat (1%), A2 |  |  |  |  |
| 19103004 | Milk, cow, fluid, reduced fat (1%), added milk solids | |  |  |  |
| 19103005 | Milk, cow, fluid, reduced fat (~1.5%), increased protein (~4%) | | |  |  |
| 19103006 | Milk, cow, fluid, lactose free, reduced fat (~1%) | |  |  |  |
| 19103007 | Milk, cow, fluid, reduced fat (1-2%), not further defined | | |  |  |
| 19103008 | Milk & water, reduced fat cow's milk & tap water | |  |  |  |
| 19104001 | Milk, cow, fluid, reduced fat (1%), added phytosterols | | |  |  |
| 19104002 | Milk, cow, fluid, reduced fat (1%), increased Ca, added Fe & vitamins C & D | | | | |
| 19104003 | Milk, cow, fluid, reduced fat (1.5%), added Ca, Mg, Zn & vitamin D | | | |  |
| 19104004 | Milk, cow, fluid, reduced fat (1.5%), added omega 3 polyunsaturates | | | |  |
| 19104005 | Milk, cow, fluid, reduced fat (1.5%), increased Ca, folate & vitamin D | | | |  |
| 19105001 | Milk, cow, fluid, skim (~0.15% fat) |  |  |  |  |
| 19105002 | Milk, cow, fluid, skim (~0.15% fat), added milk solids | |  |  |  |
| 19105003 | Milk, cow, fluid, skim (~0.15% fat), not further defined | | |  |  |
| 19105004 | Milk, cow, fluid, prepared from dry powder, skim, standard dilution | | | |  |
| 19105005 | Milk & water, skim cow's milk & tap water | |  |  |  |
| 19109002 | Milk, cow, fluid, lactose free, unfortified, not further defined | |  |  |  |
| 19109003 | Milk, cow, fluid, unflavoured, not further defined |  |  |  |  |

1. Codes for hot drinks containing cows’ milk (disaggregation of cows’ milk undertaken)

| **AFCD code** | **AFCD name** |
| --- | --- |
| 11102001 | Tea, regular, white, brewed from leaf or teabags, with cows milk not further defined |
| 11102002 | Chai latte, prepared from chai mix powder & regular fat cows milk |
| 11102003 | Chai latte, prepared from chai mix powder & reduced fat cows milk |
| 11102004 | Chai latte, prepared from chai mix powder & skim cows milk |
| 11202001 | Coffee, white, from instant coffee powder, made up with regular fat cows milk |
| 11202001 | Coffee, white, from instant coffee powder, made up with regular fat cows milk |
| 11202001 | Coffee, white, from instant coffee powder, made up with regular fat cows milk |
| 11202001 | Coffee, white, from instant coffee powder, made up with regular fat cows milk |
| 11202001 | Coffee, white, from instant coffee powder, made up with regular fat cows milk |
| 11202001 | Coffee, white, from instant coffee powder, made up with regular fat cows milk |
| 11202001 | Coffee, white, from instant coffee powder, made up with regular fat cows milk |
| 11202002 | Coffee, white, from instant coffee powder, made up with cows milk not further defined |
| 11202003 | Coffee, cappuccino, from ground coffee beans, with regular fat cows milk |
| 11202003 | Coffee, cappuccino, from ground coffee beans, with regular fat cows milk |
| 11202003 | Coffee, cappuccino, from ground coffee beans, with regular fat cows milk |
| 11202003 | Coffee, cappuccino, from ground coffee beans, with regular fat cows milk |
| 11202003 | Coffee, cappuccino, from ground coffee beans, with regular fat cows milk |
| 11202003 | Coffee, cappuccino, from ground coffee beans, with regular fat cows milk |
| 11202003 | Coffee, cappuccino, from ground coffee beans, with regular fat cows milk |
| 11202004 | Coffee, cappuccino, from ground coffee beans, double shot, with regular fat cows milk |
| 11202005 | Coffee, cappuccino, from ground coffee beans, with reduced fat cows milk |
| 11202006 | Coffee, cappuccino, from ground coffee beans, double shot, with reduced fat cows milk |
| 11202007 | Coffee, cappuccino, from ground coffee beans, with skim cows milk |
| 11202008 | Coffee, cappuccino, from ground coffee beans, double shot, with skim cows milk |
| 11202009 | Coffee, cappuccino, from ground coffee beans, with cows milk not further defined |
| 11202013 | Coffee, flat white or latte, from ground coffee beans, with regular fat cows milk |
| 11202013 | Coffee, flat white or latte, from ground coffee beans, with regular fat cows milk |
| 11202013 | Coffee, flat white or latte, from ground coffee beans, with regular fat cows milk |
| 11202013 | Coffee, flat white or latte, from ground coffee beans, with regular fat cows milk |
| 11202013 | Coffee, flat white or latte, from ground coffee beans, with regular fat cows milk |
| 11202013 | Coffee, flat white or latte, from ground coffee beans, with regular fat cows milk |
| 11202013 | Coffee, flat white or latte, from ground coffee beans, with regular fat cows milk |
| 11202014 | Coffee, flat white or latte, from ground coffee beans, double shot, with regular fat cows milk |
| 11202014 | Coffee, flat white or latte, from ground coffee beans, double shot, with regular fat cows milk |
| 11202014 | Coffee, flat white or latte, from ground coffee beans, double shot, with regular fat cows milk |
| 11202014 | Coffee, flat white or latte, from ground coffee beans, double shot, with regular fat cows milk |
| 11202014 | Coffee, flat white or latte, from ground coffee beans, double shot, with regular fat cows milk |
| 11202014 | Coffee, flat white or latte, from ground coffee beans, double shot, with regular fat cows milk |
| 11202014 | Coffee, flat white or latte, from ground coffee beans, double shot, with regular fat cows milk |
| 11202015 | Coffee, flat white or latte, from ground coffee beans, with reduced fat cows milk |
| 11202016 | Coffee, flat white or latte, from ground coffee beans, double shot, with reduced fat cows milk |
| 11202017 | Coffee, flat white or latte, from ground coffee beans, with skim cows milk |
| 11202018 | Coffee, flat white or latte, from ground coffee beans, double shot, with skim cows milk |
| 11202019 | Coffee, flat white or latte, from ground coffee beans, with cows milk not further defined |
| 11202022 | Coffee, cappuccino, flat white or latte, from ground coffee beans, double shot, with cows milk not further defined |
| 11202024 | Coffee, cappuccino, flat white or latte, from ground coffee beans, half shot, with regular fat cows milk |
| 11202024 | Coffee, cappuccino, flat white or latte, from ground coffee beans, half shot, with regular fat cows milk |
| 11202024 | Coffee, cappuccino, flat white or latte, from ground coffee beans, half shot, with regular fat cows milk |
| 11202024 | Coffee, cappuccino, flat white or latte, from ground coffee beans, half shot, with regular fat cows milk |
| 11202024 | Coffee, cappuccino, flat white or latte, from ground coffee beans, half shot, with regular fat cows milk |
| 11202024 | Coffee, cappuccino, flat white or latte, from ground coffee beans, half shot, with regular fat cows milk |
| 11202024 | Coffee, cappuccino, flat white or latte, from ground coffee beans, half shot, with regular fat cows milk |
| 11202025 | Coffee, cappuccino, flat white or latte, from ground coffee beans, half shot, with reduced fat cows milk |
| 11202026 | Coffee, cappuccino, flat white or latte, from ground coffee beans, half shot, with skim cows milk |
| 11202028 | Coffee, macchiato, from ground coffee beans, with regular fat cows milk |
| 11202028 | Coffee, macchiato, from ground coffee beans, with regular fat cows milk |
| 11202028 | Coffee, macchiato, from ground coffee beans, with regular fat cows milk |
| 11202028 | Coffee, macchiato, from ground coffee beans, with regular fat cows milk |
| 11202028 | Coffee, macchiato, from ground coffee beans, with regular fat cows milk |
| 11202028 | Coffee, macchiato, from ground coffee beans, with regular fat cows milk |
| 11202028 | Coffee, macchiato, from ground coffee beans, with regular fat cows milk |
| 11202029 | Coffee, macchiato, from ground coffee beans, with reduced fat cows milk |
| 11202030 | Coffee, macchiato, from ground coffee beans, with skim cows milk |
| 11202031 | Coffee, macchiato, from ground coffee beans, with cows milk not further defined |
| 11202033 | Coffee, mocha, from ground coffee beans, with regular fat cows milk |
| 11202033 | Coffee, mocha, from ground coffee beans, with regular fat cows milk |
| 11202033 | Coffee, mocha, from ground coffee beans, with regular fat cows milk |
| 11202033 | Coffee, mocha, from ground coffee beans, with regular fat cows milk |
| 11202033 | Coffee, mocha, from ground coffee beans, with regular fat cows milk |
| 11202033 | Coffee, mocha, from ground coffee beans, with regular fat cows milk |
| 11202033 | Coffee, mocha, from ground coffee beans, with regular fat cows milk |
| 11202034 | Coffee, mocha, from ground coffee beans, with reduced fat cows milk |
| 11202035 | Coffee, mocha, from ground coffee beans, with skim cows milk |
| 11202036 | Coffee, mocha, from ground coffee beans, with cows milk not further defined |
| 11202037 | Coffee, mocha, from ground coffee beans, double shot, with cows milk not further defined |
| 11202040 | Coffee, not further defined |
| 11202040 | Coffee, not further defined |
| 11202040 | Coffee, not further defined |
| 11202040 | Coffee, not further defined |
| 11202040 | Coffee, not further defined |
| 11202040 | Coffee, not further defined |
| 11202040 | Coffee, not further defined |
| 11204001 | Coffee, white, from instant coffee powder, decaffeinated, made up with cows milk not further defined |
| 11204002 | Coffee, cappuccino, flat white or latte, from ground coffee beans, decaffeinated, with regular fat cows milk |
| 11204002 | Coffee, cappuccino, flat white or latte, from ground coffee beans, decaffeinated, with regular fat cows milk |
| 11204002 | Coffee, cappuccino, flat white or latte, from ground coffee beans, decaffeinated, with regular fat cows milk |
| 11204002 | Coffee, cappuccino, flat white or latte, from ground coffee beans, decaffeinated, with regular fat cows milk |
| 11204002 | Coffee, cappuccino, flat white or latte, from ground coffee beans, decaffeinated, with regular fat cows milk |
| 11204002 | Coffee, cappuccino, flat white or latte, from ground coffee beans, decaffeinated, with regular fat cows milk |
| 11204002 | Coffee, cappuccino, flat white or latte, from ground coffee beans, decaffeinated, with regular fat cows milk |
| 11204003 | Coffee, cappuccino, flat white or latte, from ground coffee beans, decaffeinated, with reduced fat cows milk |
| 11204004 | Coffee, cappuccino, flat white or latte, from ground coffee beans, decaffeinated, with skim cows milk |
| 11204006 | Coffee, macchiato, from ground coffee beans, decaffeinated, with regular fat cows milk |
| 11204006 | Coffee, macchiato, from ground coffee beans, decaffeinated, with regular fat cows milk |
| 11204006 | Coffee, macchiato, from ground coffee beans, decaffeinated, with regular fat cows milk |
| 11204006 | Coffee, macchiato, from ground coffee beans, decaffeinated, with regular fat cows milk |
| 11204006 | Coffee, macchiato, from ground coffee beans, decaffeinated, with regular fat cows milk |
| 11204006 | Coffee, macchiato, from ground coffee beans, decaffeinated, with regular fat cows milk |
| 11204006 | Coffee, macchiato, from ground coffee beans, decaffeinated, with regular fat cows milk |
| 11204007 | Coffee, macchiato, from ground coffee beans, decaffeinated, with reduced fat cows milk |
| 11204008 | Coffee, mocha, from ground coffee beans, decaffeinated, with regular fat cows milk |
| 11204008 | Coffee, mocha, from ground coffee beans, decaffeinated, with regular fat cows milk |
| 11204008 | Coffee, mocha, from ground coffee beans, decaffeinated, with regular fat cows milk |
| 11204008 | Coffee, mocha, from ground coffee beans, decaffeinated, with regular fat cows milk |
| 11204008 | Coffee, mocha, from ground coffee beans, decaffeinated, with regular fat cows milk |
| 11204008 | Coffee, mocha, from ground coffee beans, decaffeinated, with regular fat cows milk |
| 11204008 | Coffee, mocha, from ground coffee beans, decaffeinated, with regular fat cows milk |
| 11204009 | Coffee, mocha, from ground coffee beans, decaffeinated, with reduced fat cows milk |
| 11801001 | Beverage, chocolate flavour, from original Milo powder, with cows milk |
| 11801003 | Beverage, chocolate flavour, from Ovaltine powder, with cows milk |
| 11803001 | Beverage, chocolate flavour, from cocoa powder, with cows milk |
| 11803002 | Beverage, chocolate flavour, from drinking chocolate, with regular fat cows milk |
| 11803003 | Beverage, chocolate flavour, from drinking chocolate, with reduced fat cows milk |
| 11803004 | Beverage, chocolate flavour, from drinking chocolate, with skim cows milk |

**Supplementary Table S2** Number of different products available and mean composition values listed on the Nutrition Information Panel of unflavoured milk-like soy, almond, oat, rice and coconut drinks sold in Australian supermarkets (Nov 2023) compared with the values in the Australian Food Composition Database (AFCD) if available

|  |  |  |  | **Protein (g/100ml)** | | **Riboflavin (mg/100ml)** | | **Vitamin B12 (µg/100ml)** | | **Iodine (µg/100ml)** | |
| --- | --- | --- | --- | --- | --- | --- | --- | --- | --- | --- | --- |
| **Type** | **Plant-based milk-like drink** | **Product details and AFCD code** | **No products** | **AFCD values** | **Mean value on products** | **AFCD values** | **Mean value on products** | **AFCD values** | **Mean value on products** | **AFCD values** | **Mean value on products** |
| Soy  27% of products available | Drink 1: Soy | 1. Regular fat, unfortified  F008721 (AFCD protein, riboflavin and iodine values derived from 10 samples in 2010, vitamin B12 values from USDA SR28,16120) | 3 | 3.8 | 3.5 | 0.031 | NI | 0 | NI | 1.4 | NI |
|  | Drink 2: Soy | 2. Regular fat, added calcium  F008719 (AFCD protein, riboflavin and iodine values derived from 10 samples in 2010, vitamin B12 values from USDA SR28,16120) | 10 | 3.8 | 3.1 | 0.031 | NI | 0 | NI | 1.4 | NI |
|  | Drink 3: Soy | 3. Regular fat, added calcium and vitamins A, B1, B2 and B12  F008720 (AFCD protein, riboflavin, vitamin B12 and iodine values derived from 10 samples in 2010) | 11 | 4.5 | 3.3 | 0.422 | 0.2 | 0.9 | 0.4 | 1.4 | NI |
|  | Other soy* | Reduced fat, added calcium, sweetened | 2 | - | 3.0 | - | NI | - | NI | - | NI |
|  | Other soy | Reduced fat, added calcium and vitamins A, B1, B2, B12 F008704 | 2 | 3.1 | 3.4 | 0.42 | 0.17 | 0.9 | 0.36 | 1.4 | NI |
|  | Other soy* | Protein plus, added calcium | 1 | - | 4.2 | - | NI | - | NI | - | NI |
|  | Other soy* | Protein plus, added calcium, B2, B12 | 1 | - | 4.0 | - | 0.3 | - | 0.4 | - | NI |

|  |  |  |  | **Protein (g/100ml)** | | **Riboflavin (mg/100ml)** | | **Vitamin B12 (µg/100ml)** | | **Iodine (µg/100ml)** | |
| --- | --- | --- | --- | --- | --- | --- | --- | --- | --- | --- | --- |
| **Type** | **Plant-based milk-like drink** | **Product details and AFCD code** | **No products** | **AFCD values** | **Mean value on products** | **AFCD values** | **Mean value on products** | **AFCD values** | **Mean value on products** | **AFCD values** | **Mean value on products** |
| Almond  34% of products available | Drink 4: Almond | 4. Added sugar, unfortified  F009825 (AFCD protein and iodine derived from 8 samples in 2018, riboflavin derived from 5 samples in 2018, B12 imputed zero) | 6 | 0.5 | 0.7 | 0.020 | NI | 0 | NI | 1.1 | NI |
|  | Drink 5: Almond | 5. Added sugar and calcium  F009827 (AFCD protein and iodine derived from 8 samples in 2018, riboflavin derived from 5 samples in 2018, vitamin B12 imputed zero) | 7 | 0.5 | 0.6 | 0.021 | NI | 0 | NI | 1.2 | NI |
|  | Drink 6: Almond | 6. Added sugar, added calcium and vitamins B1, B2 and B12  F009828 (AFCD protein and iodine derived from 8 samples in 2018, riboflavin and vitamin B12 derived from 2 samples in 2018) | 5 | 0.5 | 0.6 | 0.294 | 0.17 | 0.4 | 0.4 | 1.2 | NI |
|  | Drink 7: Almond | 7. No added sugar, added calcium  F009826 (protein and iodine derived from 8 samples in 2018, riboflavin derived from 5 samples in 2018, vitamin B12 imputed zero) | 13 | 0.5 | 0.6 | 0.021 | NI | 0 | NI | 1.2 | NI |
|  | Other almond* | No added sugar, added calcium, B1, B2, B12 | 2 | - | 0.6 | - | 0.17 | - | 0.4 | - | NI |
|  | Other almond* | No added sugar, unfortified | 2 | - | 0.7 | - | NI | - | NI | - | NI |
|  | Other almond * | Added sugar, extra creamy, added calcium | 1 | - | 2.2 | - | NI | - | NI | - | NI |
|  | Other almond* | Almond and coconut, unfortified | 1 | - | <1 | - | NI | - | NI | - | NI |
|  | Other almond* | Protein plus added calcium, B1, B2, B12 | 1 | - | 4.1 | - | 0.17 | - | 0.4 | - | NI |

|  |  |  |  | **Protein (g/100ml)** | | **Riboflavin (mg/100ml)** | | **Vitamin B12 (µg/100ml)** | | **Iodine (µg/100ml)** | |
| --- | --- | --- | --- | --- | --- | --- | --- | --- | --- | --- | --- |
| **Type** | **Plant-based milk-like drink** | **Product details and AFCD code** | **No products** | **AFCD values** | **Mean value on products** | **AFCD values** | **Mean value on products** | **AFCD values** | **Mean value on products** | **AFCD values** | **Mean value on products** |
| Oat  31% of products available | Drink 8: Oat | 8. Unfortified  F006132 (AFCD protein, riboflavin and iodine derived from 8 samples in 2015, vitamin B12 imputed zero) | 4 | 1.4 | 1.0 | 0 | NI | 0 | NI | 1.2 | NI |
|  | Drink 9: Oat | 9. Added calcium  F006131 (AFCD protein, riboflavin and iodine derived from 8 samples in 2015, vitamin B12 imputed zero) | 27 | 1.4 | 0.9 | 0 | NI | 0 | NI | 1.3 | NI |
|  | Other-oat* | Added calcium and vitamins B1, B2 and B12 | 3 | - | 0.9 | - | 0.2 | - | 0.4 | - | NI |
|  | Other oat* | Added calcium and vitamins B1, B2, B12 and iodine | 1 | - | 0.2 | - | 0.2 | - | 0.3 | - | 7.5 |
| Rice  4% of products available | Drink 10: Rice | 10. Added calcium  F007632 (AFCD protein, riboflavin and iodine derived from 12 samples in 2010, vitamin B12 imputed zero) | 5 | 0.3 | 0.5 | 0 | NI | 0 | NI | 5.4 | NI |
| Coconut  4% of products available | Drink 11: Coconut | 11. Unfortified  F009812 (AFCD protein, riboflavin and iodine derived from 8 samples in 2018, vitamin B12 imputed zero) | 4 | 0.2 | 0.3 | 0 | NI | 0 | NI | 1.3 | NI |

NI No Information listed on packet, ingredient list indicates no fortification; *No composition values for this product in the AFCD, Shading indicates plant-based milk-like drinks used in dietary modelling

**Supplementary Table S3** The proportion of total protein, riboflavin, vitamin B12 and iodine intake provided by cows’ milk (including cows’ milk consumed in hot drinks e.g. tea, coffee and hot chocolate) in the ‘Base case’

|  | **Percentage of total intake provided by cows’ milk** | | | |
| --- | --- | --- | --- | --- |
|  | **Protein  (%)** | **Riboflavin (%)** | **Vitamin B12 (%)** | **Iodine  (%)** |
| **Males** |  |  |  |  |
| 2-3 years | 18.5 | 33.9 | 50.4 | 45.8 |
| 4-8 years | 10.7 | 22.0 | 35.5 | 28.2 |
| 9-13 years | 9.0 | 21.4 | 30.9 | 26.7 |
| 14-18 years | 7.0 | 18.8 | 23.6 | 22.2 |
| 19-30 years | 6.0 | 16.4 | 20.6 | 20.8 |
| 31-50 years | 6.9 | 19.1 | 24.1 | 23.3 |
| 51-70 years | 6.6 | 18.7 | 22.3 | 21.6 |
| 71 years and over | 7.8 | 20.1 | 24.7 | 22.7 |
| **Females** |  |  |  |  |
| 2-3 years | 17.3 | 32.3 | 47.7 | 41.6 |
| 4-8 years | 10.4 | 21.1 | 34.9 | 26.8 |
| 9-13 years | 9.3 | 21.5 | 31.9 | 25.2 |
| 14-18 years | 6.2 | 15.9 | 22.1 | 19.0 |
| 19-30 years | 7.1 | 17.6 | 25.3 | 22.2 |
| 31-50 years | 7.9 | 20.8 | 28.2 | 24.3 |
| 51-70 years | 7.6 | 19.7 | 24.9 | 23.2 |
| 71 years and over | 8.9 | 22.7 | 28.4 | 26.2 |
|  |  |  |  |  |
